# Adaptive swarm cluster-based dynamic multi-objective synthetic minority oversampling technique algorithm for tackling binary imbalanced datasets in biomedical data classification

**DOI:** 10.1186/s13040-016-0117-1

**Published:** 2016-12-01

**Authors:** Jinyan Li, Simon Fong, Yunsick Sung, Kyungeun Cho, Raymond Wong, Kelvin K. L. Wong

**Affiliations:** 1 Department of Computer and Information Science, University of Macau, Taipa, Macau, S.A.R. China; 2 Computer Engineering Division, Keimyung University, Daegu, South Korea; 3 Department of Multimedia Engineering, College of Engineering, Dongguk University, Dongdaeipgu, South Korea; 4 School of Computer Science and Engineering, University of New South Wales, Sydney, NSW 2000 Australia; 5 Centre for Biomedical Engineering, School of Electrical & Electronic Engineering, University of Adelaide, Adelaide, Australia; 6 School of Medicine, Western Sydney University, Campbelltown, Sydney Australia

**Keywords:** Imbalanced dataset, Swarm optimisation, Under-sampling, SMOTE, Dynamic Multi-objective, Classification, Biomedical data

## Abstract

**Background:**

An imbalanced dataset is defined as a training dataset that has imbalanced proportions of data in both interesting and uninteresting classes. Often in biomedical applications, samples from the stimulating class are rare in a population, such as medical anomalies, positive clinical tests, and particular diseases. Although the target samples in the primitive dataset are small in number, the induction of a classification model over such training data leads to poor prediction performance due to insufficient training from the minority class.

**Results:**

In this paper, we use a novel class-balancing method named adaptive swarm cluster-based dynamic multi-objective synthetic minority oversampling technique (ASCB_DmSMOTE) to solve this imbalanced dataset problem, which is common in biomedical applications. The proposed method combines under-sampling and over-sampling into a swarm optimisation algorithm. It adaptively selects suitable parameters for the rebalancing algorithm to find the best solution. Compared with the other versions of the SMOTE algorithm, significant improvements, which include higher accuracy and credibility, are observed with ASCB_DmSMOTE.

**Conclusions:**

Our proposed method tactfully combines two rebalancing techniques together. It reasonably re-allocates the majority class in the details and dynamically optimises the two parameters of SMOTE to synthesise a reasonable scale of minority class for each clustered sub-imbalanced dataset. The proposed methods ultimately overcome other conventional methods and attains higher credibility with even greater accuracy of the classification model.

## Background

Machine learning plays an important role in knowledge discovery and automatic recognition in biomedical applications. Specifically, classification is a machine learning technique that integrates the complex relationships between the input variables and the target classes of some biomedical data. Automatic pattern recognition and prediction are then possible with the learnt model when unseen data are tested. Machine learning from biomedical data encounters several difficulties, mainly because these datasets are characterised by incompleteness (missing values), incorrectness (collection error or noise in the data), inexactness (data retrieved from incorrect sources) and sparseness (too few records available). Another subtle problem that transcends the integrity of the data is an imbalanced class distribution; that is, there are too few target data that the users are interested in amongst too much ordinary data collected. For instance, some decision support systems in health care applications deal with patient data that include very few positive records in a large population, especially for new diseases. Other examples are cancer genes in microarrays [1], abnormal sub-sequences in biosignal patterns [2], tiny cysts in mammograms of the biological imaging field [3] and the colony distribution and mutation of *E. coli* or yeast [4, 5], in addition to classification in the biomedical engineering field [6], etc.

The imbalanced dataset problem is known to cause pseudo-accuracy – a spuriously good prediction rate with low credibility. A classification model that is learnt from a majority of mediocre data becomes biased towards the majority class and less sensitive to recognition of the minority class samples [7]. Testing this classifier with the same training dataset shows a high prediction accuracy on the surface. However, when the model is tested with new unseen samples of the minority class, the accuracy rate plummets, which indicates that the falsely high accuracy of the training model is futile and unreliable.

The current approach of rebalancing the imbalanced dataset is to simply inflate the population of the minority class by randomly copying its data or shrinking the amount of majority class data until they match the population of the other class. This approach works by matching the populations of the classes merely in quantity. And when it comes to repeated experiment for more than ten times, this approach ignores the subtle underlying mappings between the input variables and the target classes, which can be highly nonlinear. In a nutshell, adjusting the quantity of data from each class to the same level does not guarantee generation of the most effective classifier. The methods used to attain a balanced dataset such as the aforementioned over-sampling [8] and under-sampling [9], both of which change the numbers of two classes’ samples. These methods are at the data level. Furthermore, there also exist some techniques at the algorithm level to overcome the imbalanced problem in classification. Cost-sensitive learning [10] is a commonly used method in which distinct weights are dispatched to the two classes to pressurise the classifier to the minority class. Boosting methods [11, 12] include many weak classifiers to obtain a strong classifier to avoid the imbalance problem. All combinations of class distributions were attempted with a support vector machine (SVM) as a performance measure [13].

Our proposed algorithm is based on the classical version of the Synthetic Minority Oversampling Technique (SMOTE) [14], which is the most popular and effective method to rebalance the original dataset and conquer the imbalance problem. Its basic idea is to allow the algorithm to fabricate extra minority data into the dataset by observing and assessing the characteristics of the minority class sample’s spatial structure. We assume an over-sampling rate of *N* (equation (1) synthesises *N* times new minority class samples) and each minority class sample *x*_*i*_ ∈*S*_*minority*_. The other parameter *k* is used by the algorithm to examine *k* neighbours of *x*_*i*_ in the minority class samples, and then to randomly select *x*_*t*_ from the *k* neighbours by using equation (1) to generate the synthetic data *x*_*new*_ [15]:

1 $$ {x}_{new}={x}_i+\left({x}_t-{x}_i\right)\times {v}_{rand}, $$

where *v*_*rand*_ is a random number between 0 and 1, and *N* and *k* are the two important parameters of this algorithm that are used to generate the suitable number and characteristic samples of the minority class.

We adopt particle swarm optimization (PSO) [16] to search for optimal values for the pair of parameters for SMOTE. PSO is a widely used meta-heuristic algorithm that imitates the feeding process of birds. Assuming a population *X* = (*X*_1_, *X*_2_,…, *X*_*n*_) that is grouped by *n* particles in a *D* dimensional search space, the *i* ^th^ particle in this space is expressed as a vector *X*_*i*_ with *D* dimension, *X*_*i*_ = (*x*_*i*1,_ *x*_*i*2_, …, *x*_*iD*_)^*T*^, and the position of the *i* ^th^ particle in the search space represents a potential solution that is coded as a combination of the parameters values of *K* and *S* for SMOTE. As an objective function, the program can calculate the corresponding fitness of position *X*_*i*_ of each particle, where the speed of the *i* ^th^ particle is *V*_*i*_ = (*V*_*i*1_,*V*_*i*2_, …, *V*_*iD*_)^*T*^, the extremum value of each agent is *P*_*i*_ = (*P*_*i*1_, *P*_*i*2_, …, *P*_*iD*_)^*T*^ and the extremum of the population is *P*_*g*_ = (*P*_*g*1_, *P*_*g*2_, …, *P*_*gD*_)^*T*^. During the process of iteration, the extremum values of each agent and the population will update their positions and speeds. Equations (2) and (3) show the mathematical process as follows:

2 $$ {V}_{id}^{t+1} = \omega {V}_{id}^t + {c}_1{r}_1\left(\ {P}_{id}^t - {X}_{id}^t\right) + {c}_2{r}_2\left(\ {P}_{gd}^t - {X}_{id}^t\right), $$

3 $$ {X}_{id}^{t+1}={X}_{id}^t + {V}_{id}^{t+1}\ . $$

In Equation (2), *ω* is the inertial weight; *d* = 1, 2, …, *D*; *i* = 1, 2, …, *n*; *t* is the current iteration time; *c*_1_ and *c*_2_ are non-negative constants as the velocity factor, *r*_1_ and *r*_2_ are random values between 0 to 1 and *V*_*id*_ is the particle speed.

Our proposed approach introduces under-sampling and ensemble techniques to controllably cluster majority class samples into several sub-majority class datasets, which will respectively combine the original minority class dataset to generate the corresponding sub-datasets. The imbalanced sub-datasets will then make use of PSO to determine their suitable parameters for SMOTE for the over-sampling operation and finally obtain the average of their results. In addition to the accuracy of the classification model, the Kappa value is another objective used to assure the robustness and credibility in our experiment. Therefore, during the process of searching for our approach, we also solve a dynamic multi-objective problem. Compared with other methods, the proposed methods could combine different previous skills together and attain leap ascension of the classification credibility under the premise of maintaining high accuracy.

## Methods

In classification, especially classification of a flawed dataset, the only indicator of accuracy is not persuasive. Even though it may be sharp, the results will still lead to misleading judgments and testing. The supplementary parameters used to measure and distinguish the classification model of imbalanced datasets are receiver operating characteristic area [17], F-measure (abbreviated as F-1) [18] and G-mean [19]. In this paper, we collect the F-measure and G-mean as our reference parameters. The Kappa statistic [20] is another favourable assessment index used to effectively estimate the credibility of the classification model. In the imbalanced dataset’s classification, the low Kappa value accompanied a high level of accuracy because most classification algorithms neglected the minority class samples and misclassified them in the majority class. The target class commonly takes a very small percentage in quantity; thus the number of misclassified minority class samples produces a low error rate. As a result, the precision of the trained model will encounter a serious crisis of confidence when it meets multiple target class samples in a testing dataset. However, the low Kappa statistic will directly present the credibility of the classification.

For this reason, the second objective of Kappa is implemented in our experiment to intuitively and objectively represent the consistency of the results and the reliability of classification. The theoretical range of Kappa is between −1 and 1. There are six intervals or degrees of interpretation of a Kappa outcome from −1 to 1 [21]: Kappa < 0, less than chance agreement; 0.01 ≤ Kappa ≤ 0.20, slight agreement; 0.21 ≤ Kappa ≤ 0.40, fair agreement; 0.41 ≤ Kappa ≤ 0.60, moderate agreement; 0.61 ≤ Kappa ≤ 0.80, substantial agreement; 0.81 ≤ Kappa ≤ 1.00, almost perfect agreement. In our previous papers [22, 23], we adopted the other interpretation for Kappa to split the area into four parts with values of 0, 0.4 and 0.75, which respectively presented the meaning of meaningless, low credibility, general credibility and strong credibility. In our experiment, we have mentioned that in order to guarantee the precision and credibility of the classification mode, both accuracy and the Kappa value were our targets, which caused dynamic changes in the values. The optimisation algorithm is Swarm, the intelligence algorithm is PSO, and the assistant verification algorithm is Neural Network, which will cooperate with each other to find two suitable and best parameters (*N* of over-sampling rate and *k* neighbours) of SMOTE to synthesise the minority samples and the variation in the class distribution to remit the imbalance problem. Equations (4) and (5) are our fitness functions, which are calculated from the confusion matrix.

4 $$ Accuracy = \frac{TP+TN}{P+N}, $$

5 $$ Kappa=\frac{P_o - {P}_c}{1 - {P}_c}, $$

6 $$ {P}_o= Accuracy = \frac{TP+TN}{P+N}, $$

7 $$ {P}_c = \frac{\left(TP+FP\right)\times \left(TP+FN\right)+\left(FN+TN\right)\times \left(FP+TN\right)}{{\left(P+N\right)}^2}. $$

Note that TP, TN, FP, and FN, respectively represent true positive, true negative, false positive and false negative. P stands for positive and N for negative. Po and Pc are the measures of the percentage of agreement and the chance of agreement respectively. Neural Network is used to estimate and verify the fitness of each iteration of the PSO. Figure 3 presents a snapshot of the fluctuation patterns of accuracy and kappa as the transformation progress (from TP = 0, TN = 0, FP = 100, FN = 5 to TP = 100, TN = 5, FP = 0, FN = 0) of a confusion matrix in an imbalanced dataset classification model, G-mean and F-measure as the auxiliary metrics. In this example, there are 100 majority class samples and 5 minority class samples. At the 606th cycle of iterations, accuracy and Kappa both have reached a very high value of approximately 1. Since the two objectives are not opposing each other, a special type of optimization called the non-inferior set tactics [24] is adopted here and customized for this specific rebalancing task. Furthermore, it shows Kappa is more sensitive than the commonly used metrics of G-mean and F-measure to judge the bias of the imbalanced classification model from the confusion matrix.

The classification results are evaluated by different training and testing parts. We perform a tenfold cross validation [25, 26] to test the corresponding performance of the current dataset classification model. That means the dataset randomly is divided into ten parts averagely, and each part will take turns being the testing dataset with the other nine parts as training datasets in the repeated ten times’ classifications. The Kappa, Accuracy, G-mean and other performances of this cross-validation process are averaged from these ten classifications. Moreover, to keep the fairness of the experiment, each dataset tested Random SMOTE, SRA and proposed methods separately ten times, and the final results pertain to the mean value of the experiments.

**Fig. 1:**
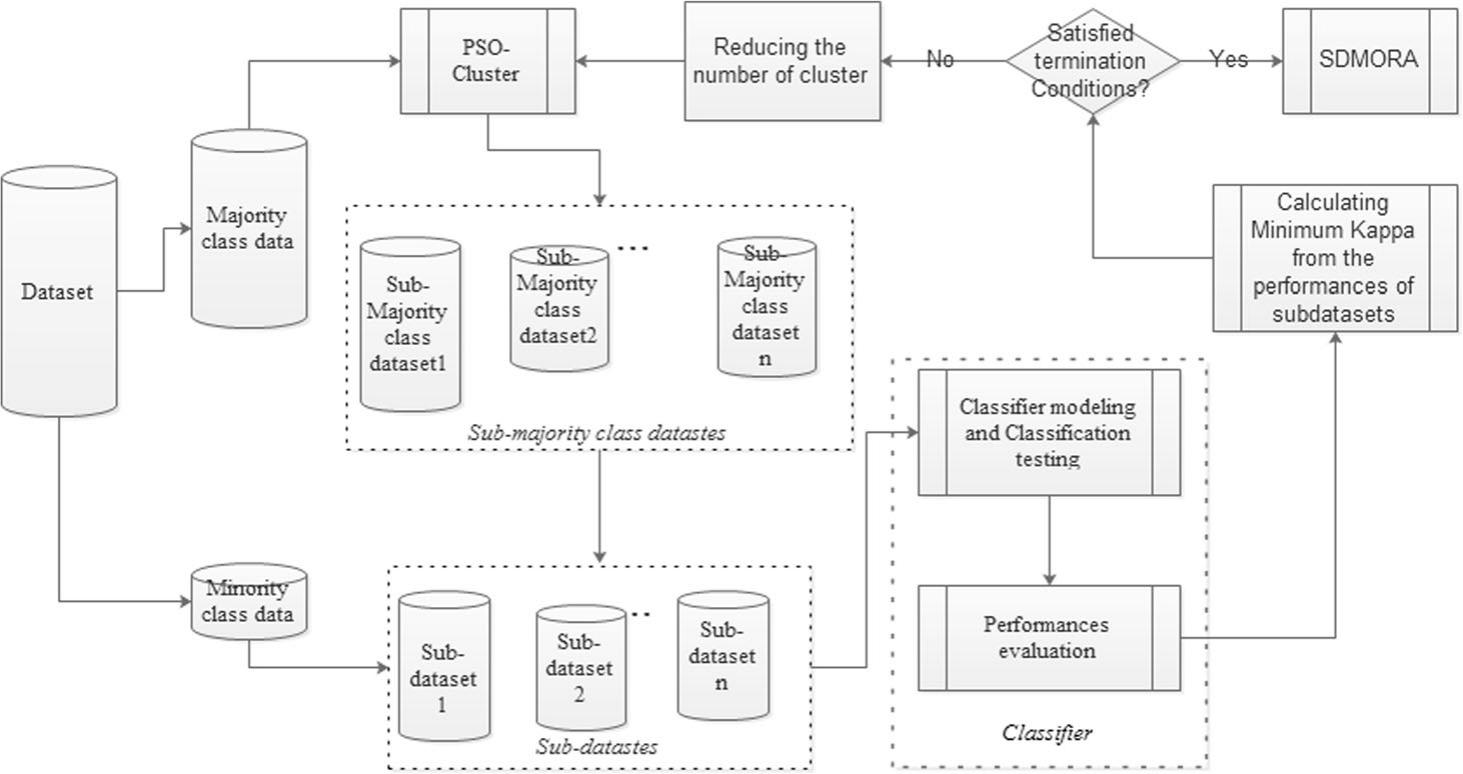
Principle of adaptive swarm clustered-based dynamic multi-objective synthetic minority oversampling technique (SMOTE)

The reason for combining cluster under-sampling and over-sampling lies in the detailed grouping of the majority dataset. For instance, if the intelligent medical diagnostic system only records and divides the collected data into two classes - gastric cancer data and other data, then there is no doubt that non-cancer cases comprise the vast majority of the whole. These samples contain many different situations, such as gastritis, gastric ulcer, gastric peroration and healthy. Hence, the cluster of non-cancer data can include more detailed illness diagnoses and narrow the imbalance ratio of the original dataset. As under-sampling and over-sampling, the proposed algorithm can be divided into two parts. Figure 1 shows the principle diagram of this algorithm, and this paper also provides the pseudo code in the below to describe the operation process.

In the first part, the dataset is divided into majority class data and minority class data, which will be processed respectively. PSO optimized *k*-means clusters algorithm [27] the majority class into several categories as a strategy, and each sub-majority class dataset is combined with the minority class dataset to establish the corresponding sub-dataset, which will be reprocessed by the second part to perform over-sampling separately. The *k*-means algorithm is a widely used algorithm for cluster in data mining [28]. It randomly select *k* instances as the center of *k* classes, and according to the Euclidean distance, the rest instances will be respectively assigned to the closest class. Then this process will be repeated until the sum of squared error of the centre converge. Thus the initial defended value of *k* and the center of classes for *k*-means directly impact the cluster effect. PSO has strong global searching ability which helps k-means to avoid falling local-best. Since internal information sharing between particles in the population in each iteration, the results converge rapidly and steadily. The fitness function of PSO optimized k-means cluster algorithm adopts the Euclidean distance as its fitness function to find out the appropriate center of the classes. Moreover, compared with the previous methods [29, 30], in order to find the global best solution in this step, there are two termination conditions assisting PSO to obtain a reasonable *k* value of *k*-means (where *k* is the number of clusters). The first condition is that the number of clusters must be greater than one, and the second is that the minimum value of Kappa in all of the classification results of the numerous sub-datasets must be greater than 0.2. Here, the classifier still implements the Neural Network. Therefore, PSO can assist *k*-means adaptively find out the proper centre of classes and the value of *K* to overcome weakness of the traditional *k*-means algorithm. Furthermore, in a new sub-dataset, if the original minority class samples overcome the other class samples in quantity, Neural Network will directly classify this sub-dataset. Otherwise this dataset will perform the over-sampling operation.

The second part is the evolutionary version of the Swarm Rebalancing Algorithm (SRA) [17, 22], which is called the Swarm Dynamic Multi-objective Rebalancing Algorithm (SDMRA) or DMSMOTE. This algorithm is used instead of SRA to fix a credible value of Kappa to promote accuracy. The final result is the average value of all sub-datasets, as shown in Equation (8).

8 $$ final\kern0.5em  result=\left({\displaystyle {\sum}_1^c}\left({p}_n\right)\right)/c. $$

**Fig. 2:**
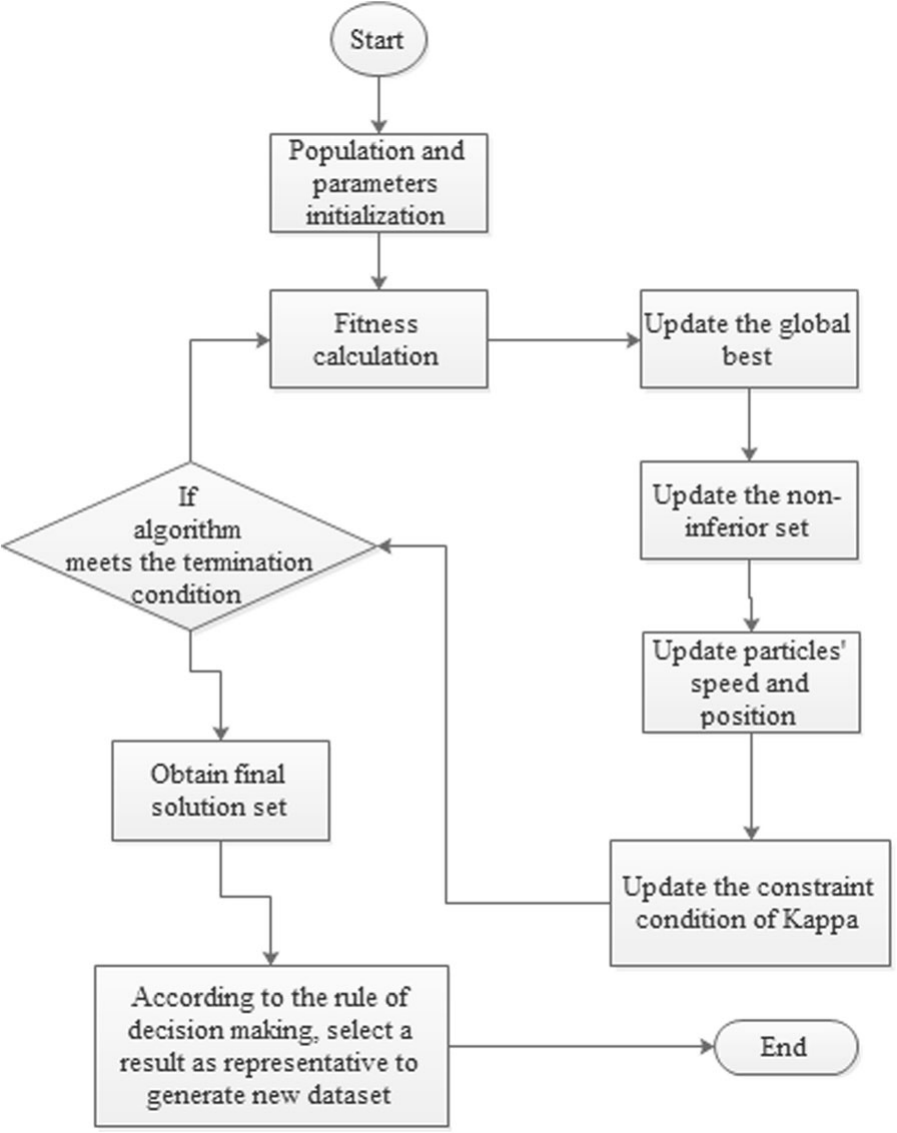
Flow chart of dynamic multi-objective SMOTE (SDMRA)


In Equation (8), *p*_*n*_ stands for all performances (Kappa, Accuracy, G-mean, F-measure, etc.) of each sub-dataset and *c* is the number of clusters. Figure 2 is the flow chart that presents our algorithm’s second part. The concept of non-inferior [24] sets was adopted in the PSO to solve the dynamical multi-objective problem. In the initial step, algorithm filters and produces the non-inferior set; filtering will input a particle that is not controlled by the others into the non-inferior set, which will casually offer a solution as the global best before the particles update. Then, because the new particles are not handled by the other particles and the particles non-inferior set, these particles will be input into the non-inferior set to update it. Meanwhile, a particle will be randomly selected from the non-inferior set as the global best before swarm renewal. During the process of iteration, the particles’ update criteria include accuracy and Kappa of the older particle that are worse than the new; one of accuracy and Kappa of the new particle is better than the older one, and the absolute value of the other index’s difference of the new and older is smaller than the defined tolerance; the Kappa value is smaller than the current threshold value of Kappa (0.2, 0.4, 0.6 or 0.8). When a new particle satisfies any criteria, it will replace the older one, or this position will be randomly removed. Because the results in the non-inferior set are commonly more than one, we select the solution whose product of Kappa and accuracy is the best as the final result of this sub-dataset. Meanwhile, we name the product as having reliable accuracy for this improved performance.

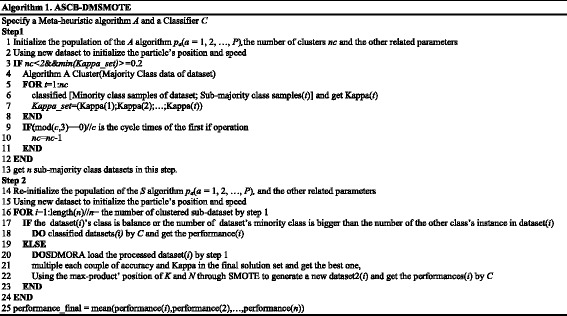


Direct Neural Network, SMOTE + Neural Network, Random-SMOTE [31], and our forgoing version of SRA (PSO SMOTE) + Neural Network constitute the comparison benchmark. We generate the completely balanced dataset with SMOTE. Random-SMOTE is used to randomly pick out parameters for SMOTE to generate a new dataset, using the average of ten times of Random-SMOTE as the ultimate result of each dataset. PSO SMOTE (SRA) has two update conditions, based on the requirements that Kappa will be greater than the fixed Kappa threshold (0.4), and that the accuracy must be greater than that of the previous position. In the experiments, the populations of the PSO and the maximum iteration are 20 and 100, respectively. Note that Matlab (version 2014) has been utilized to code and compile the whole program, and the operating and computing environment for all experiments is in the workstation with CPU: CPU: E5-1650 V2 @ 3.50 GHz, RAM: 32 GB.

The target of our experiment is the binary classification problem. We obtained our seven biomedical or bioinformatics datasets from Ding [32] and UCI [33]. Some multi-class datasets were also modified by Ding to be binary classes as the target is needed or to take the tiny proportion. Table 1 presents information on the datasets. The Imb.Ratio is the ratio between the majority class and the minority class, ranging from 5.7:1 to 42:1. The target indicates the minority class in the dataset. The selected dataset contains the clinic dataset of thoracic surgery, disease datasets of thyroid sick and Arrhythmia; the biological image dataset of mammography; and the microbiological dataset of E. coli and yeast. Thus it can be seen that the imbalanced dataset appeared in different orientations of the biologic domain.

**Table 1:** Information of our biomedical datasets

Name	Imb.Ratio	Target	Samples
Thoraric Surgery	5.7:1	died	470
Ecoli	8.6:1	imU	336
Sick Euthyroid	9.8:1	sick euthyroid	3163
Yeast_ML8	13:1	target 8	2407
Thyroid Sick	15:1	sick	3772
Arrhythmia	17:1	class = 06	452
Mammography	42:1	minority	11183

## Results and discussion

**Fig. 3:**
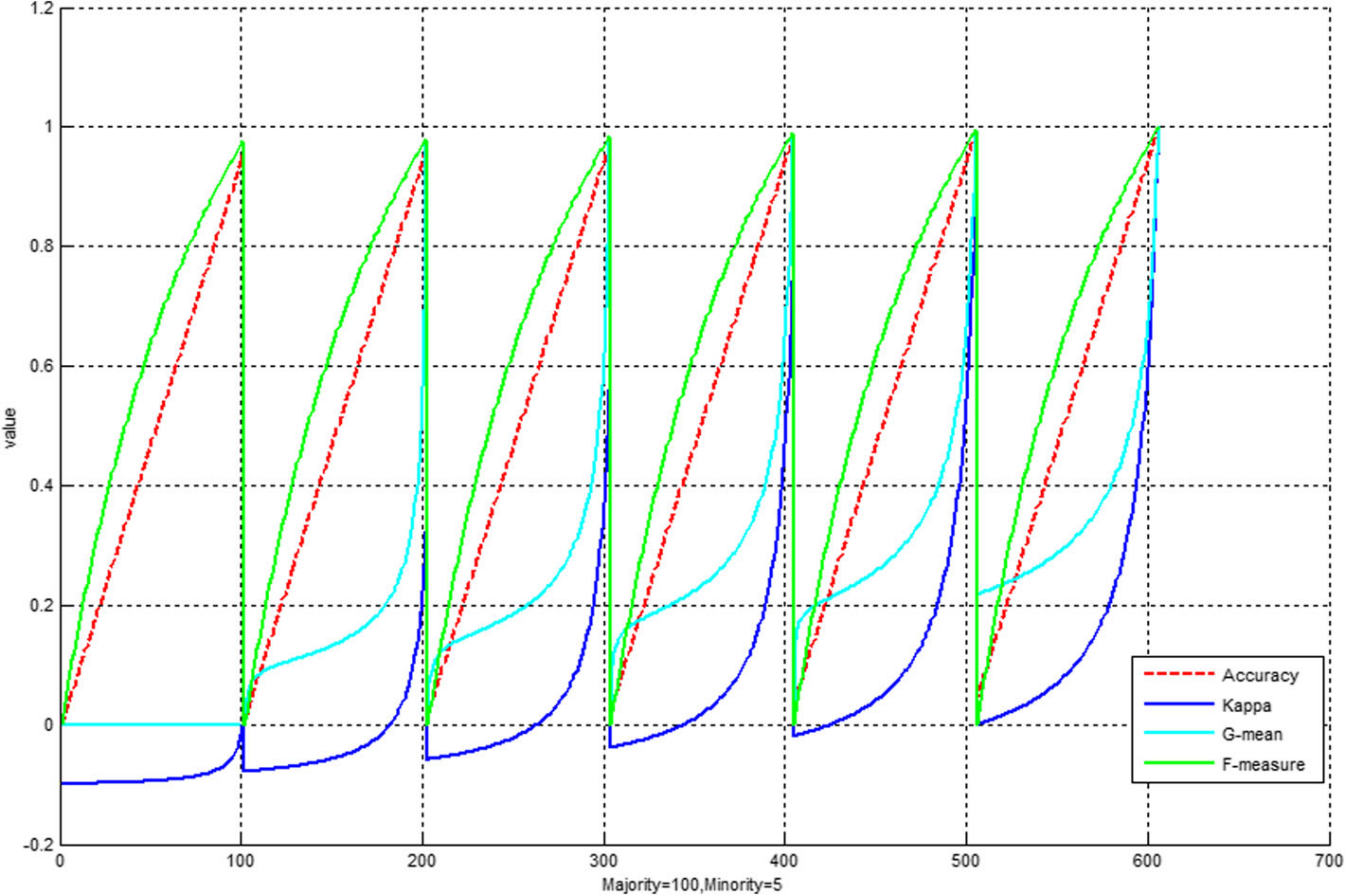
Snapshot of fluctuating values of accuracy and Kappa (an example of imbalanced dataset with 100 majority class samples and 5 minority class samples)

**Fig. 4:**
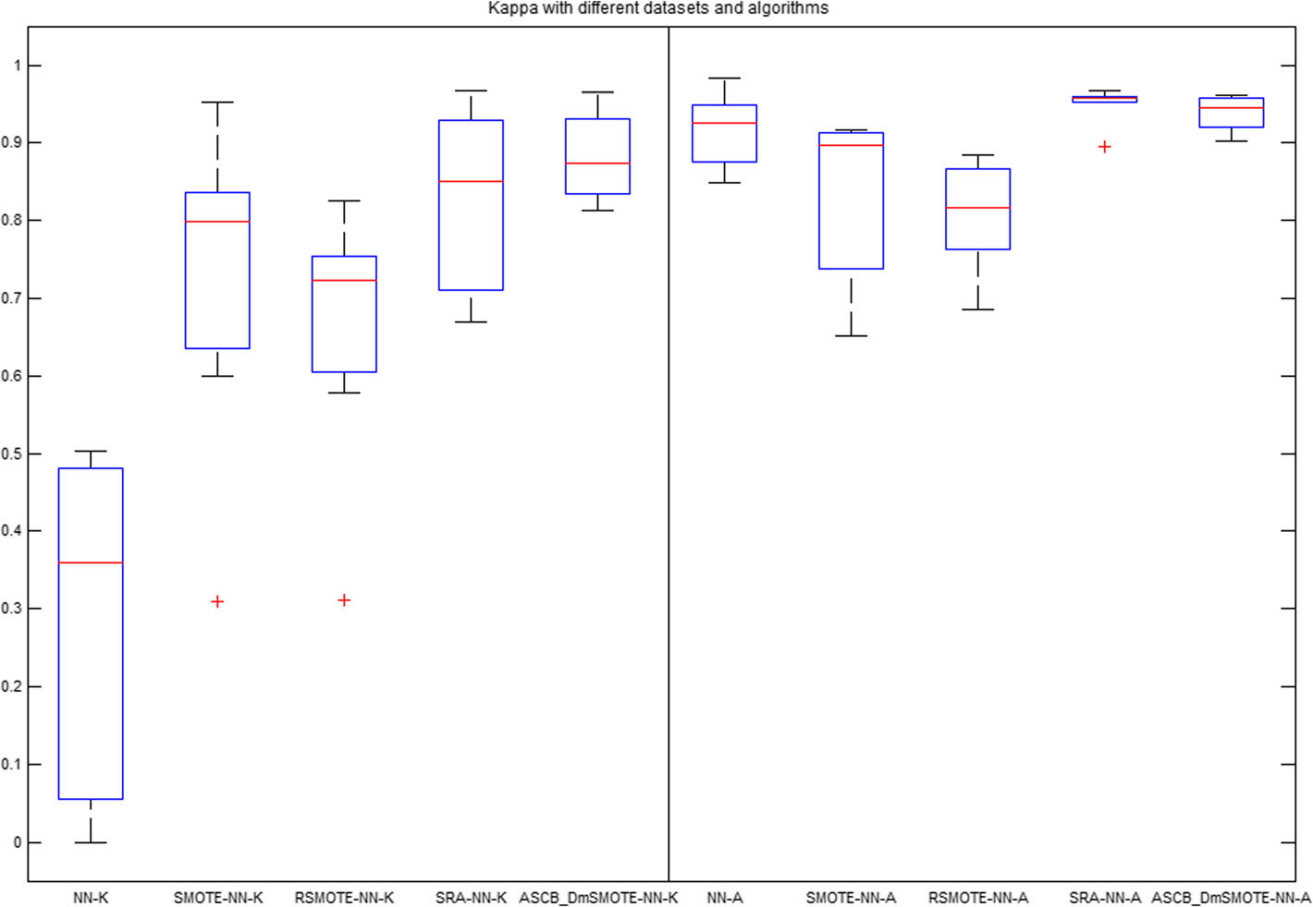
Average Kappa and Average Accuracy of different methods over all datasets in boxplot

**Fig. 5:**
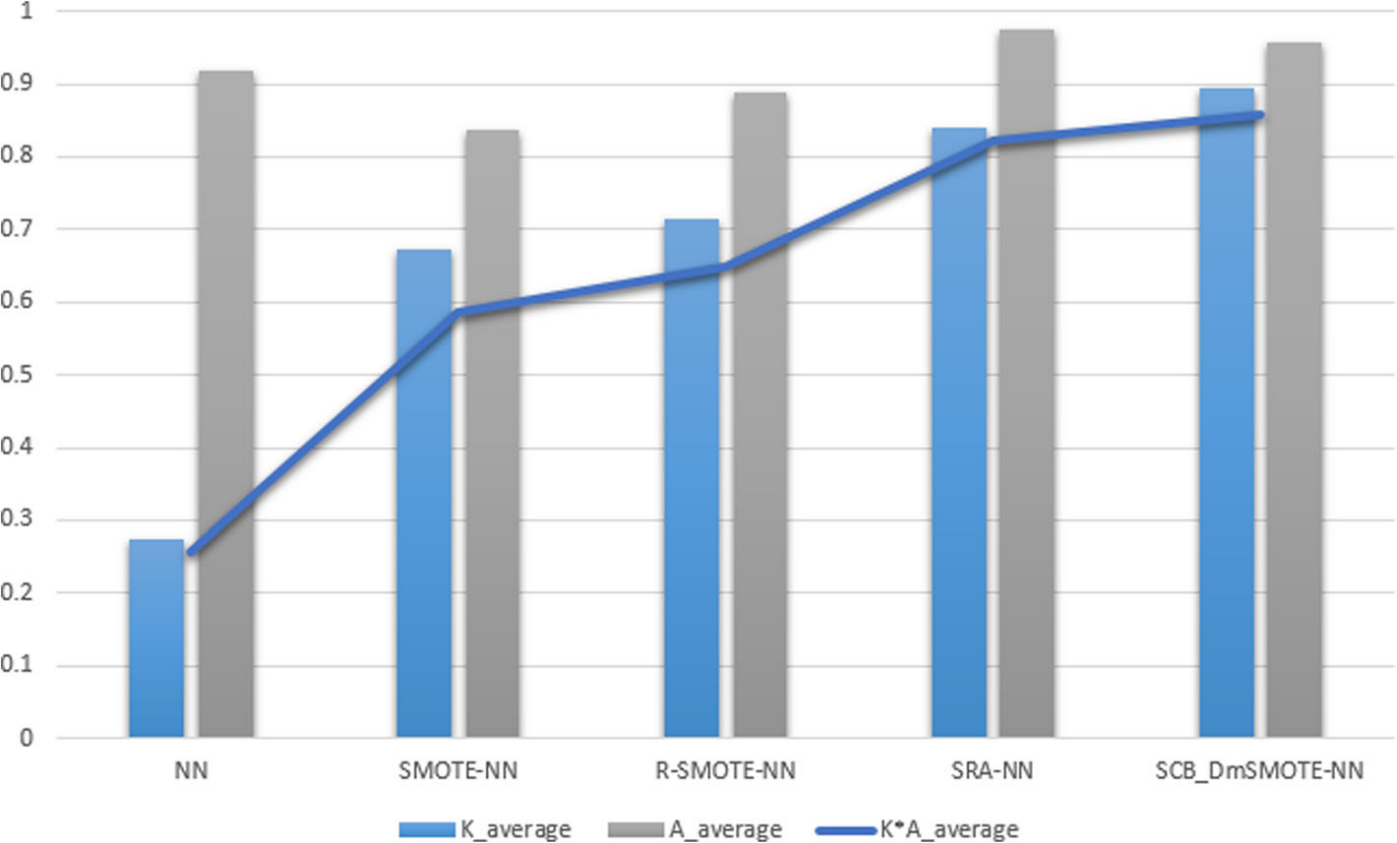
Average of Kappa, Accuracy and Reliable accuracy of all experiments

**Fig. 6:**
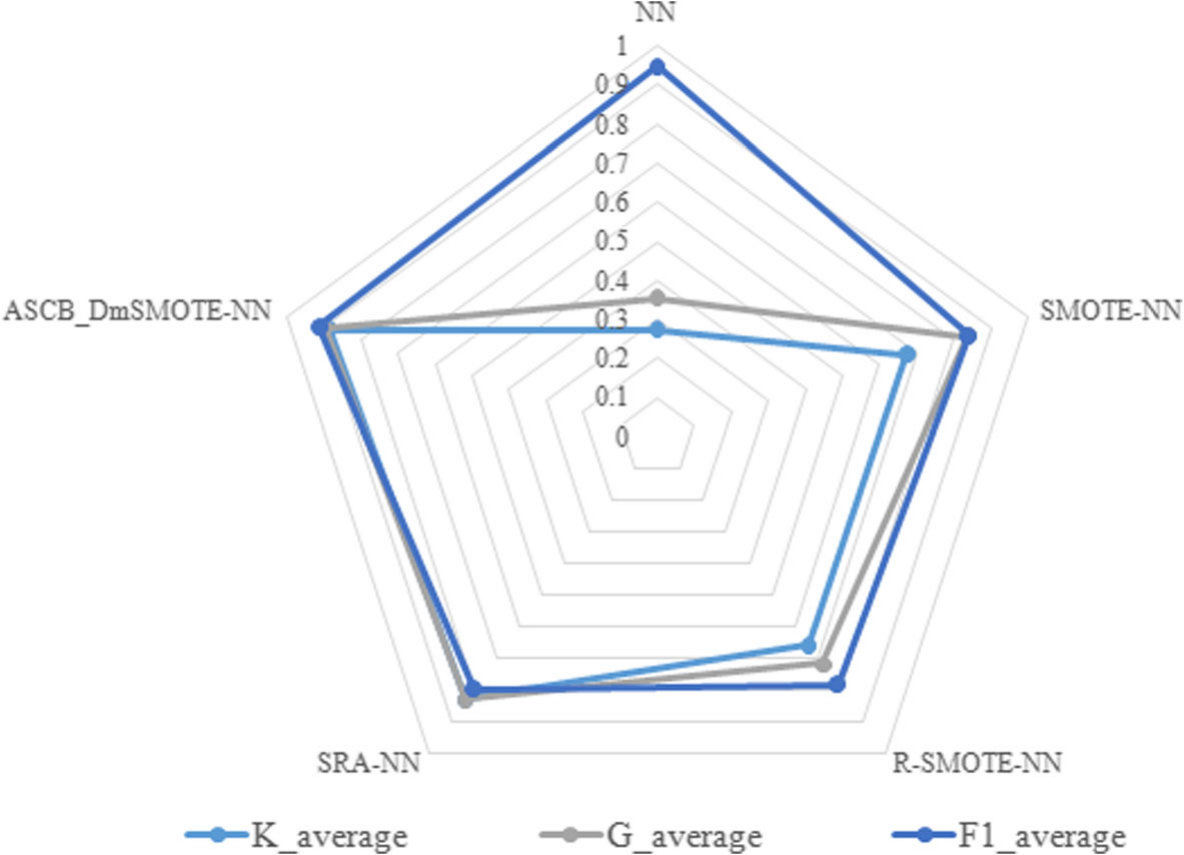
Average performance of imbalanced dataset classification indices of all experiments

**Fig. 7:**
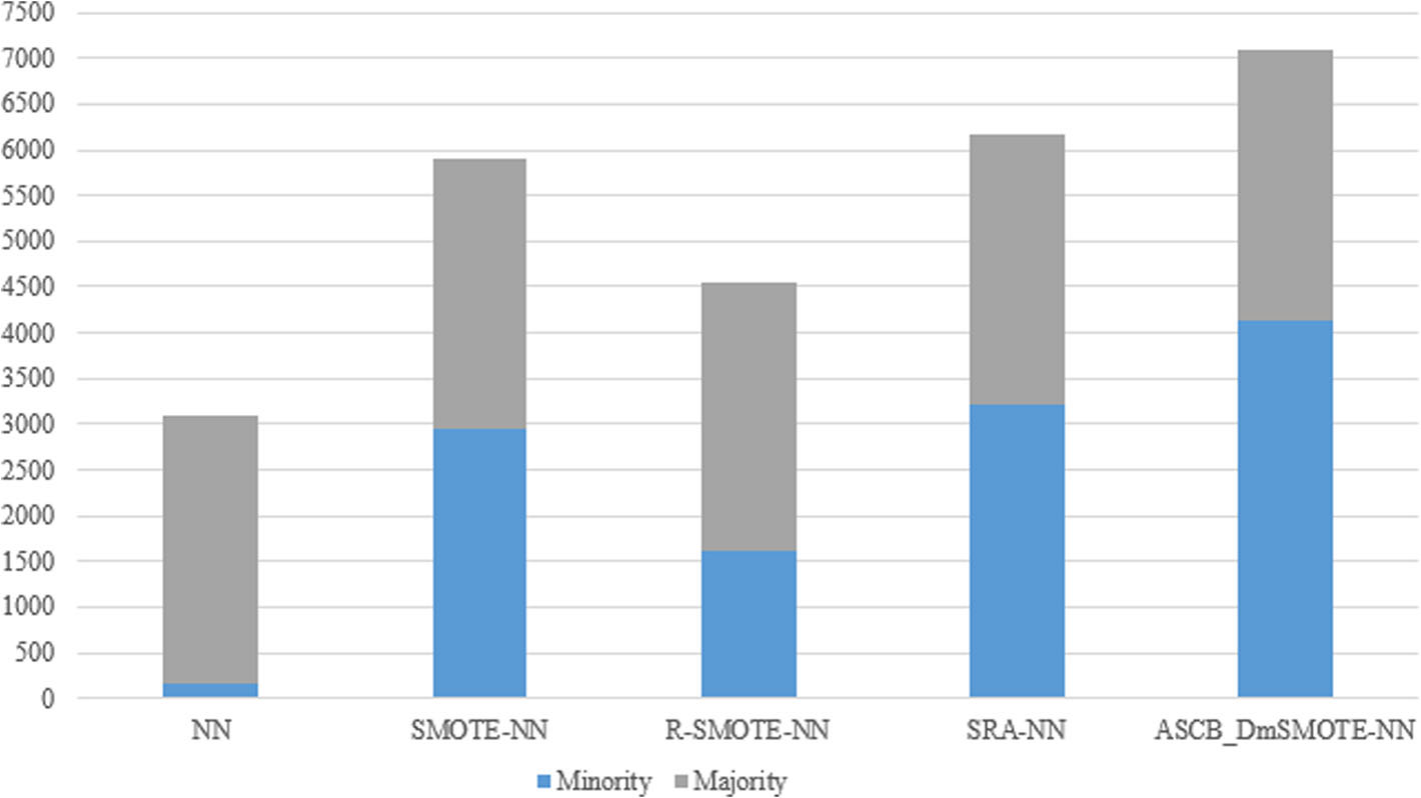
Size variations of datasets with different methods processed

The average performance in terms of Kappa, accuracy, G-mean, F-measure, and Imbalanced ratio as well as their respective offsets are shown in Tables 2, 3, 4, 5 and 6, respectively. The bold values significantly indicate the best value for each dataset and method. Meanwhile, the averages of these indicators are visualized in Figs. 3, 4, 5, 6 and 7 for comparison of the capabilities and variations of the different approaches. All figures and tables include NN, SMOTE-NN, R-SMOTE-NN, SRA-NN and ASCB-DMSMOTE-NN on behalf of Neural Network, SMOTE with Neural Network, Random-SMOTE with Neural Network, PSO-SMOTE with Neural Network and the proposed Adaptive Swarm Clustered Based Dynamic Multi-objective SMOTE.

**Table 2:** Average Kappa of different algorithms with different datasets

Kappa	NN	SMOTE-NN	R-SMOTE-NN	SRA-NN	ASCB_DmSMOTE-NN
Thoraric Surgery	0.049	0.305	0.312 ± 0.48	0.670 ± 0.21	*0.813* ± 0.11
Ecoli	0.502	0.807	0.723 ± 0.12	0.850 ± 0.05	*0.848* ± 0.06
Sick Euthyroid	0.497	0.831	0.688 ± 0.13	0.824 ± 0.07	*0.874* ± 0.05
Yeast_ML8	*0.000*	0.381	0.578 ± 0.23	*0.968* ± 0.02	0.927 ± 0.04
Thyroid Sick	0.360	0.833	0.762 ± 0.12	*0.906* ± 0.06	0.829 ± 0.11
Arrhythmia	0.068	0.761	0.826 ± 0.13	0.937 ± 0.04	*0.966* ± 0.02
Mammography	0.436	0.794	0.729 ± 0.14	0.673 ± 0.16	*0.932* ± 0.02
K_average	0.273	0.673	0.660 ± 0.22	0.833 ± 0.09	*0.884* ± 0.06

The italicized entries represent the best performance

**Table 3:** Average Accuracy of different algorithms with different datasets

Accuracy	NN	SMOTE-NN	R-SMOTE-NN	SRA-NN	ASCB_DmSMOTE-NN
ThoraricSurgery	0.848	0.653	0.686 ± 0.23	0.895 ± 0.05	*0.902* ± 0.03
Ecoli	0.925	0.904	0.817 ± 0.18	*0.959* ± 0.03	0.918 ± 0.02
Sick Euthyroid	0.936	0.916	0.781 ± 0.19	*0.952* ± 0.03	0.927 ± 0.02
Yeast_ML8	0.926	0.690	0.756 ± 0.18	*0.968* ± 0.04	0.959 ± 0.02
Thyroid Sick	0.953	0.916	0.852 ± 0.13	*0.961* ± 0.03	0.946 ± 0.03
Arrhythmia	0.858	0.880	0.871 ± 0.11	0.958 ± 0.04	*0.961* ± 0.03
Mammography	*0.983*	0.897	0.884 ± 0.10	*0.960* ± 0.03	0.956 ± 0.02
A_average	0.919	0.837	0.807 ± 0.16	*0.950* ± 0.03	0.938 ± 0.02

The italicized entries represent the best performance

**Table 4:** Average G-mean value of different algorithms with different dataset

G-mean	NN	SMOTE-NN	R-SMOTE-NN	SRA-NN	ASCB_DmSMOTE-NN
ThoraricSurgery	0.179	0.651	0.479 ± 0.22	0.715 ± 0.12	*0.843* ± 0.05
Ecoli	0.630	0.904	0.768 ± 0.18	0.813 ± 0.12	*0.875* ± 0.04
Sick Euthyroid	0.613	0.916	0.750 ± 0.15	0.832 ± 0.10	*0.916* ± 0.04
Yeast_ML8	0.000	0.690	0.641 ± 0.14	0.926 ± 0.07	*0.928* ± 0.05
Thyroid Sick	0.453	0.916	0.811 ± 0.14	*0.898* ± 0.05	0.836 ± 0.6
Arrhythmia	0.091	0.880	0.802 ± 0.13	0.904 ± 0.06	*0.951* ± 0.4
Mammography	0.520	0.896	0.795 ± 0.12	0.746 ± 0.06	*0.926* ± 0.5
G_average	0.355	0.836	0.721 ± 0.14	0.833 ± 0.10	*0.896* ± 0.05

The italicized entries represent the best performance

**Table 5:** Average F-measure of different algorithms with different datasets

F-measure(F1)	NN	SMOTE-NN	R-SMOTE-NN	SRA-NN	ASCB_DmSMOTE-NN
ThoraricSurgery	*0.917*	0.667	0.643 ± 0.27	0.642 ± 0.1	0.865 ± 0.04
Ecoli	*0.959*	0.902	0.762 ± 0.18	0.795 ± 0.09	0.874 ± 0.04
Sick Euthyroid	*0.966*	0.916	0.793 ± 0.16	0.787 ± 0.07	0.891 ± 0.05
Yeast_ML8	*0.962*	0.693	0.809 ± 0.15	0.902 ± 0.09	0.939 ± 0.04
Thyroid Sick	*0.976*	0.915	0.820 ± 0.15	0.863 ± 0.08	0.821 ± 0.03
Arrhythmia	0.876	0.878	0.847 ± 0.16	0.895 ± 0.08	*0.952* ± 0.04
Mammography	*0.991*	0.901	0.812 ± 0.13	0.726 ± 0.07	0.943 ± 0.03
F1_average	*0.949*	0.839	0.784 ± 0.17	0.801 ± 0.09	0.912 ± 0.04

The italicized entries represent the best performance

**Table 6:** Average Imbalanced ratio (majority: minority) value of different algorithms with different datasets

Imb.Ratio (ma/mi)	NN	SMOTE-NN	R-SMOTE-NN	SRA-NN	ASCB_DmSMOTE-NN
ThoraricSurgery	5.7:1	1:1	1.2 ± 0.7:1	0.7 ± 0.4:1	0.5 ± 0.3:1
Ecoli	8.6:1	1:1	1.3 ± 0.5:1	0.6 ± 0.2:1	0.4 ± 0.3:1
Sick Euthyroid	9.8:	1:1	1.8 ± 0.5:1	1.1 ± 0.3:1	0.7 ± 0.4:1
Yeast_ML8	12.6:	1:1	1.9 ± 0.6:1	0.6 ± 0.2:1	0.7 ± 0.2:1
Thyroid Sick	15.3:1	1:1	1.6 ± 0.4:1	0.8 ± 0.3:1	0.9 ± 0.1:1
Arrhythmia	17.1:1	1:1	1.3 ± 0.7:1	0.7 ± 0.3:1	0.5 ± 0.2:1
Mammography	42.0:1	1:1	1.5 ± 0.5:1	0.9 ± 0.2:1	0.8 ± 0.3:1
I_average	15.9:1	1:1	1.5 ± 0.6:1	0.8 ± 0.3:1	0.6 ± 0.3:1

Figure 4 illuminates the results of our two targets, Kappa and accuracy, and the left 5 boxes stand for Kappa value with the symbol ‘-K’ and the right 5 boxes indicate accuracy with the symbol ‘-A‘. These two box plots are homologous to Tables 7 and 2. We can observe that the average Kappa of NN of the original dataset is lower than 0.4 and that the length of the box is great. Especially in the Yeast_ML8 dataset, the minimum Kappa value is zero but its accuracy is greater than 0.9 – a typical pseudo-high accuracy as mentioned above which has no credibility. The original SMOTE totally rebalances the imbalanced dataset. The second boxes of Kappa show great increases in the average and the height of the body. However, SMOTE sacrifices accuracy to obtain credibility. In contrast, the performances of Random-SMOTE are more stable than those of SMOTE, even though its median Kappa value is less than that of SMOTE. PSO-SMOTE has the highest accuracy and a higher Kappa value than the previous three. Moreover, it is the most significant because it improves the Kappa value and promotes the accuracy of the best value of 1. The accuracy of the proposed algorithm is slightly lower than that of PSO-SMOTE, but the Kappa is higher; furthermore, the length of the boxes shows that the proposed approach is more settled than our previous version and that there is no discrete point on the whole.

**Table 7:** List of abbreviations

Abbreviation	Meaning	Page
ASCB_DmSMOTE	Adaptive Swarm Cluster-Based Dynamic Multi-objective SMOTE	1
SDMRA/DMSMOTE	Swarm Dynamic Multi-objective Rebalancing Algorithm	6
Imb.Ratio	Imbalanced Ratio	7
ma/mi	Majority class/Minority class	13
NN	Neural Network	7
PSO	Particle Swarm Optimization	3
R-SMOTE	Random -SMOTE	7
SMOTE	Synthetic Minority Oversampling Technique	1
SRA	Swarm Rebalancing Algorithm	6

As mentioned above, we created and introduced an index called reliable accuracy which was the product of Kappa and accuracy. Kappa represents the degree of the classification model’s agreement, reliability and credibility; thus we can connect these two indicators to assess the accuracy in truth. In addition, this is also a strategy of decision making to select a suitable pair of solutions from the non-inferior set. Figure 5 presents the average kappa, accuracy and reliable accuracy of each method. The results of the line diagram agree with those of the above discussions about the two box plots. Through the radar chart of Fig. 6, we compare the three commonly used auxiliary evaluation fingers. In our experiment, F-measure (F1) almost lost its effect. We note that G-mean and Kappa have nearly the same consistent variation even though Kappa is more sensitive and cautious.

The last bar diagram of Fig. 7 reveals the variations of the minority class data from the majority class data. With reference from Table 6, we find that our methods synthesise many minority class samples, even the number of minority class is more than the number of majority class in the new dataset, which renders our methods to require more time for processing, as shown in Fig. 6. However, their performance is better, and this also illustrate that the absolute equilibrium distribution of classes does not pertain to the best results.

## Conclusions

In this paper, our proposed approach, ASCB_DmSMOTE, can overcome the imbalanced dataset problems in biomedical classification. It reasonably re-allocates the majority class in the details and dynamically optimises the two parameters of SMOTE to synthesise a reasonable scale of minority class for each sub-dataset and ultimately attains higher credibility of the classification model and even greater accuracy. This algorithm is a new version of SMOTE, and through the swarm intelligence algorithm, our swarm rebalancing series of algorithm can effectively combine the over-sampling, under-sampling and ensemble techniques. In addition to such a combination of methods, they can also be used with the population’s path to consecutively determine the best and most reasonable global solution. The new concept of reliable accuracy not only deals with decision making but also can be more direct and valid to evaluate a classification model. Its performances are much steadier than those of the previous version of our algorithms. Furthermore, it is able to more scientifically and effectively generate better and more reasonable synthetic data than the traditional class rebalancing algorithm. This work offers insights to biomedical practitioners who consider the application of computational tools to subside the imbalanced dataset problem, which is typically inherently in biomedical data.
